# The effect of reparixin on survival in patients at high risk for in-hospital mortality: a meta-analysis of randomized trials

**DOI:** 10.3389/fimmu.2022.932251

**Published:** 2022-07-25

**Authors:** Giovanni Landoni, Alberto Zangrillo, Gioia Piersanti, Tommaso Scquizzato, Lorenzo Piemonti

**Affiliations:** ^1^ Department of Anesthesia and Intensive Care, Istituti di Ricovero e Cura a Carattere Scientifico (IRCCS) San Raffaele Scientific Institute, Milan, Italy; ^2^ Faculty of Medicine, Vita-Salute San Raffaele University, Milan, Italy; ^3^ Diabetes Research Institute, Istituti di Ricovero e Cura a Carattere Scientifico (IRCCS) San Raffaele Scientific Institute, Milan, Italy

**Keywords:** CXCR2 antagonist, Reparixin, CXCL-8, intensive & critical care, COVID-19, SARS-CoV-2

## Abstract

**Introduction:**

A great number of anti-inflammatory drugs have been suggested in the treatment of SARS-CoV-2 infection. Reparixin, a non-competitive allosteric inhibitor of the CXCL8 (IL-8) receptors C-X-C chemokine receptor type 1 (CXCR1) and C-X-C chemokine receptor type 2 (CXCR2), has already been tried out as a treatment in different critical settings. Due to the contrasting existing literature, we decided to perform the present meta-analysis of randomized controlled trials (RCTs) to investigate the effect of the use of reparixin on survival in patients at high risk for in-hospital mortality.

**Methods:**

We created a search strategy to include any human RCTs performed with reparixin utilization in patients at high risk for in-hospital mortality, excluding oncological patients. Two trained, independent authors searched PubMed, EMBASE, and the Cochrane Central Register of Controlled Trials (CENTRAL) for appropriate studies. Furthermore, references of review articles and included RCTs were screened to identify more studies. No language restrictions were enforced. To assess the risk of bias of included trials, the Revised Cochrane risk-of-bias tool for randomized trials (RoB 2) was used.

**Results:**

Overall, six studies were included and involved 406 patients (220 received reparixin and 186 received the comparator). The all-cause mortality in the reparixin group was significantly lower than that in the control group [5/220 (2.3%) in the reparixin group vs. 12/186 (6.5%) in the control group, odds ratio = 0.33 (95% confidence interval 0.12 to 0.96), *p*-value for effect 0.04, *p* for heterogeneity 0.20, *I* ^2^ = 36%]. In addition, no difference in the rate of pneumonia, sepsis, or non-serious infections was shown between the two groups.

**Conclusion:**

Our meta-analysis of randomized trials suggests that short-term inhibition of CXCL8 activity improved survival in patients at high risk for in-hospital mortality without increasing the risk of infection.

**Meta-analysis registration:**

PROSPERO, identifier CRD42021254467.

## Introduction

The coronavirus disease 2019 (COVID-19) pandemic, caused by the severe acute respiratory syndrome coronavirus 2 (SARS-CoV-2), made it necessary to repurpose existing drugs (1). Since an amplified inflammatory response leading to an uncontrolled cytokine release can be a consequence of SARS-CoV-2 infection, several anti-inflammatory drugs (glucocorticoids, non-steroidal anti-inflammatory drugs, interleukin antagonists, and kinase inhibitors) are being evaluated to be repositioned in COVID-19 (2).

Immediately after the start of the pandemic, we had suggested reparixin (also called repertaxin), a non-competitive allosteric inhibitor of the CXCL8 (IL-8) receptors C-X-C chemokine receptor type 1 (CXCR1) and C-X-C chemokine receptor type 2 (CXCR2), as a potentially effective molecule after the successful treatment of four patients with extremely severe COVID-19 (3). The rationale for using reparixin lay in the hypothesis that the systemic and autocrine IL-8–CXCR-1/-2 axis was at the center of neutrophil-driven immunopathology in severe COVID-19. In fact, neutrophil activation is a key pathophysiological feature of the systemic inflammatory response; while neutrophils play a protective role against invading pathogens, their unrestrained activation may lead to tissue injury associated with the release of cytotoxic neutrophil extracellular traps (NETs) (4, 5). Subsequent data confirmed our initial hypothesis (6–13). Elevated levels of NETs, neutrophilia, high neutrophil-to-lymphocyte ratio (NLR), neutrophil activators (CXCL8/IL-8 and granulocyte colony-stimulating factor), and effectors (resistin, the IL-8-inducer lipocalin-2, and hepatocyte growth factor) have been reported as indicators of severe respiratory disease and poor outcomes in COVID-19 patients (14–20). As further confirmation, the administration of reparixin in patients with severe COVID-19 pneumonia improved clinical outcomes and facilitated respiratory recovery in 56 patients enrolled in an open-label, randomized, phase 2 study (21).

Before and independently of the COVID-19 outbreak, evidence was provided in support of the involvement of exaggerated pro-inflammatory activation of neutrophils, accompanied by the release of cytotoxic NETs, in the pathogenesis of clinical derangements present in critically ill patients (4, 22–24). High NLR, neutrophil percentage-to-albumin ratio (NPAR), neutrophil-to-albumin ratio (NAR), neutrophil-derived enzyme myeloperoxidase, IL-8, and NETs have been reported as indicators of severe disease and poor outcomes in cardiogenic and septic shock, in acute lung injury (ALI) and acute respiratory distress syndrome (ARDS), in disseminated intravascular coagulation, and in acute kidney injury (22, 25–42).

Since several RCTs on the use of reparixin patients at high risk for in-hospital mortality were published, we decided to perform a systematic review and meta-analysis of RCTs to investigate the effect of reparixin on survival in these patients.

## Method

### Search strategy and study selection

Two trained, independent investigators searched PubMed, EMBASE, ClinicalTrials.gov, and the Cochrane Central Register of Controlled Trials (CENTRAL) for appropriate studies. Due to the paucity of studies, the full search strategies simply included the word reparixin or repertaxin. We selected any RCTs ever performed with reparixin in patients at high risk for in-hospital mortality with the exclusion of oncological settings. Furthermore, we contacted international experts and applied backward snowballing to retrieve additional manuscripts (i.e., looking through references of an identified set of articles and reviews).

At first, two investigators independently examined references at a title or abstract level with divergences resolved by mediation of a third author. Relevant references were collected as complete articles.

The inclusion criteria used for potentially pertinent studies were random allocation to treatment (reparixin vs. any comparator without restrictions on dose or time of administration) and studies involving at high risk for in-hospital mortality (patients were considered at high risk for in-hospital mortality if they had at least one organ dysfunction and/or were receiving intensive care or emergency treatments at the time of randomization). The exclusion criteria were oncological settings and non-adult patients. Compliance to selection criteria was assessed by two independent investigators and studies were selected for the final analysis. Divergences were resolved by consensus. Searches are updated on 26 April 2022.

### Data extraction

Two investigators individually retrieved data on baseline, procedure, and outcome. They extracted data following the intention-to-treat principle whenever possible. In case of missing data, they contacted the corresponding authors *via* e-mail. The primary endpoint of the present review was mortality rate at the longest available follow-up. Secondary endpoints were the risk of getting pneumonia, of having sepsis, and the occurrence of a non-serious infection.

### Assessment of risk of bias

The risk of bias of randomized studies was appraised according to the Revised Cochrane risk-of-bias tool for randomized trials (RoB 2) (43), and divergences were resolved by consensus. Publication bias was evaluated with visually inspecting funnel plots.

### Data analysis

The meta-analysis was accomplished using Review Manager software (RevMan, version 5.4. Copenhagen: The Nordic Cochrane Centre, The Cochrane Collaboration, 2020).

The odds ratio (OR) with a 95% confidence interval (CI) was calculated for dichotomous variables, whereas the risk ratio (RR) with a 95% CI was calculated for common events, defined as the frequency of the event occurring in the control group being >10%. We calculated the proportion of patients with the outcome in each group, and the *p*-value for the comparison between the groups. A *p*-value ≤ 0.05 was considered statistically significant. In addition, we also calculated the number needed to treat (NNT).

Heterogeneity was explored using *I*² statistic and the *χ*² test, with significance being set at *p*-values of 0.10. A fixed-effects model for the meta-analysis was used in the presence of low heterogeneity, defined as *I*² result < 50% and a *p*-value > 0.10 in the *χ*² test. If significant heterogeneity was identified, defined as a *p*-value of ≤0.10, we employed a random-effects model, unless one or two trials were found to dominate the available evidence, or significant publication bias was present.

Sensitivity analyses were performed by analyzing the data with a fixed-effects model versus a random-effects model, and changing the summary statistics (ORs, risk differences, or RRs) or by removing each study in turn.

We performed a fixed-effects model trial sequential analysis with an overall type I error of 5% and a power of 80%. We hypothesized a 20% relative risk reduction (RRR) and a mortality of 10% in the control arm. The meta-analysis monitoring boundaries, required information size (RIS), diversity-adjusted information size (*D* ^2^), and adjusted 95% confidence intervals were quantified. All data analyses were performed with R version 3.6.1, except trial sequential analysis using TSA software version 0.9.5.10.

This study was registered on PROSPERO (CRD 42021254467) and performed in compliance with The Cochrane Collaboration and Preferred Reporting Items for Systematic Reviews and Meta-Analyses guidelines.

## Results

Database searches, contacts with experts, and snowballing yielded a total of 13 articles (**Supplementary Figure 1**). Six studies were excluded because of our prespecified exclusion criteria: one was not randomized (44), two did not involve patients at high risk for in-hospital mortality (45, 46), two included cancer patients (47, 48), and one was conducted in healthy volunteers (49). A sixth study (50) was excluded because it was a *post-hoc* analysis of a single-center small cohort derived from a multicentric trial (46). The six manuscripts included in the present meta-analysis (21, 51–55) randomized 406 patients (220 received reparixin and 186 received the comparator).

### Trials’ characteristics

Studies were conducted in North America and Europe and were published from 2008 to 2022 (**Table 1**). Trials were performed in solid organ transplant recipients (three trials, 215 patients) (51, 53, 54), in patients with severe chronic or recurrent acute pancreatitis undergoing total pancreatectomy with islet autotransplantation (55), in patients undergoing on-pump coronary artery bypass grafting (52), and in patients with severe COVID-19 pneumonia (21) (one trial each with 102, 32 and 55 patients, respectively). The most frequently used dose was an intravenous infusion of 2.8 mg/kg/h, and the most frequently used length of infusion was 1 week (**Table 2**). The most frequent control treatment was placebo (four trials, 149 patients), while the other two trials used standard care as control (**Table 2**).

**Table 1:** Description of the studies included in the meta-analysis, in order of year of publication.

First author	Year	Journal	Setting	Country (the first is of the corresponding author)	Number of patients in the reparixin group	Number of patients in the control group
Meyers BF	2008	*J Heart Lung Transplant*	Primary graft dysfunction in lungs transplantation	USA, Canada, Italy	46	55
Opfermann P	2015	Clin Exp Immunol	Ischemia–reperfusion injury and inflammation after on-pump coronary artery bypass graft surgery	Austria	16	16
Zhuravel SG	2017	ClinicalTrial.gov	Orthotopic liver transplantation	Russian Federation and Belarus	22	18
Remuzzi G	2020	ClinicalTrial.gov	Ischemia–reperfusion injury kidney transplantation	Italy, USA, France and Spain	48	26
Witkowski P	2021	*Am J Transplant*	Pancreatectomy for chronic pancreatitis	USA, Canada, Italy	52	52
Landoni G	2022	*Infect Dis Ther*	COVID-19	Italy	36	19

**Table 2:** Doses and modalities of administration of reparixin in the eight included randomized studies.

First author	Posology (mg/kg/h)	Intravenous or orally	Comparator	Length of treatment	Total administered dose	Length of follow-up
Meyers BF	2.8 mg/kg/h	Intravenous	Placebo	48 h	134.4 mg/kg	1 year
Opfermann P	4.5 mg/kg/h for 30 min followed by continuous infusion at 2.8 mg/kg/h until 8 h after the end of CPB	Intravenous	Placebo	8 h	24.7 mg/kg	90 days
Zhuravel SG	2.8 mg/kg/h	Intravenous	Standard care	7 days	470.4 mg/kg	1 year
Remuzzi G	Variable doses	Intravenous	Placebo	<1 day	27–33.3 mg/kg	365 ± 14 days
Witkowski P	2.8 mg/kg/h administered at 0.25 ml/kg/h	Intravenous	Placebo	7 days	498 mg/kg	365 ± 14 days
Landoni G	3,600 mg/day	Orally	Standard care	7 days	25,200 mg	7 days

### Quantitative data synthesis

#### Primary endpoint

**Figure 1:**
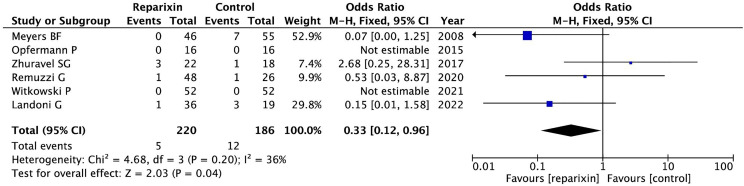
Forest plot of the effect of reparixin on mortality according to the six included randomized studies.

**Figure 1** shows the forest plot of the effect of reparixin on mortality according to the six included randomized studies. Thus, mortality of patients treated with reparixin was significantly lower than mortality in controls: 5/220 (2.3%) in the reparixin group vs. 12/186 (6.5%) in the control group, OR = 0.33 [95% CI 0.12 to 0.96], *p* for effect 0.04, *p* for heterogeneity 0.20, *I* ^2^ = 36%, number needed to treat = 24.

Magnitude and direction of findings were maintained in all sensitivity analyses including those that removed one study in turn (**Supplementary Figure 2**) and in subgroup perioperative settings (**Supplementary Figure 3**), transplant patients (**Supplementary Figure 4**), and length of treatment ≥48 h (**Supplementary Figure 5**).

Overall, risk of bias analysis showed that four included studies were at low risk of bias (accounting for 311 patients), and two trials were considered at unclear risk of bias (95 patients) (**Supplementary Figure 6**).

**Figure 2:**
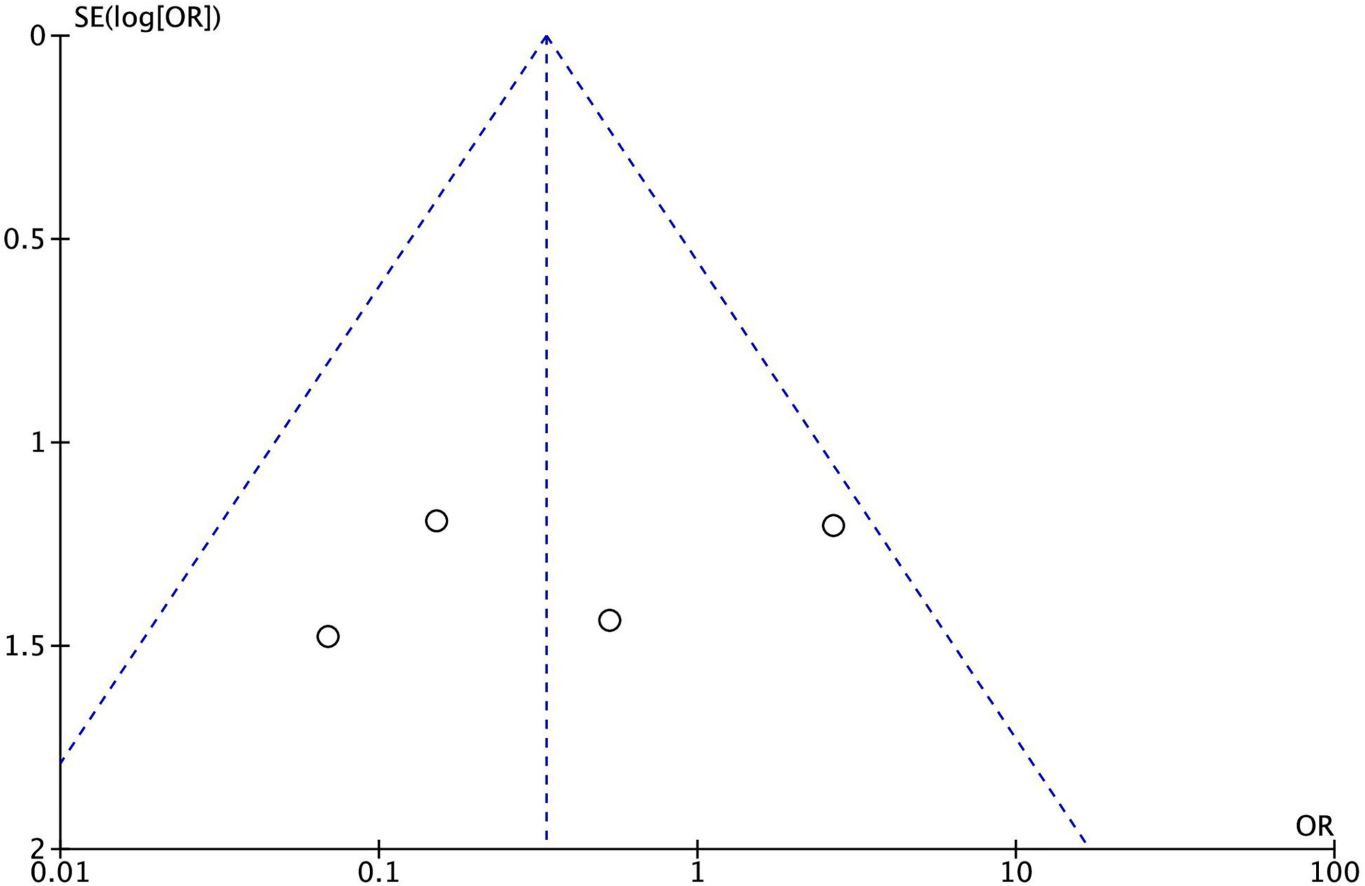
Funnel plot of the effect of reparixin on mortality according to the six included randomized studies.

The funnel plot did not reveal the presence of small study bias (**Figure 2**). Moreover, trial sequential analysis (OR = 0.33; trial sequential analysis-adjusted 95% CI 0.12–0.96; *p* = 0.04; *I* ^2^ = 36%) did not indicate that our findings are conclusive. The cumulative Z-curve did not cross the monitoring boundary curve for benefit and did not reach the required information size (*n* = 742) (**Supplementary Figure 7**).

Mortality reduction was confirmed in the subgroups of perioperative, transplant, and length of treatment ≥48 h (**Supplementary Figures 1–3**).

#### Secondary endpoint

**Figure 3:**
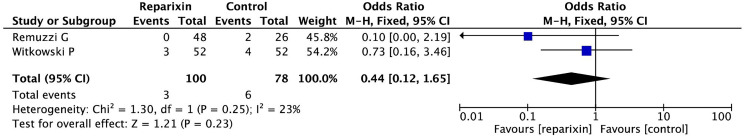
Forest plot of the effect of reparixin on developing pneumonia.

**Figure 4:**
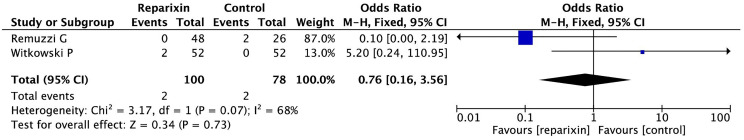
Forest plot of the effect of reparixin on developing sepsis.

**Figure 5:**
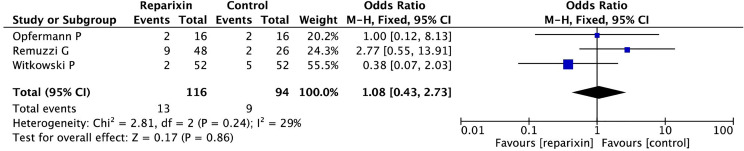
Forest plot of the effect of reparixin on the occurrence of a non-serious infection.

We found no difference in the rate of pneumonia (3 of 100 [3.0%] in the reparixin group vs. 6 of 78 [7.7%] in the control group, OR = 0.44 [95% CI 0.12–1.65], *p* for effect = 0.23, *I* ^2^ = 23%, two trials included; see **Figure 3**), sepsis (2 of 100 [2.0%] in the reparixin group vs. 2 of 78 [2.6%] in the control group, OR = 0.76 [95% CI 0.16–3.56] *p* for effect = 0.73, *I* ^2^ = 68%, two trials included; see **Figure 4**), and non-serious infections (13 of 116 [11.2%] in the reparixin group vs. 9 of 94 [9.6%] in the control group, OR = 1.08 [95% CI 0.43–2.73] *p* for effect = 0.86, *I* ^2^ = 29%, three trials included; see **Figure 5**) between reparixin and controls.

## Discussion

In this meta-analysis of randomized studies, we found that reparixin, a non-competitive allosteric inhibitor of CXCR1 and CXCR2, is associated with better survival in patients at high risk for in-hospital mortality. This was associated with a similar rate of infective complications. Chemokine receptors are relevant therapeutic targets for the treatment of many human diseases; indeed, more than 100 experimental chemokine receptor antagonists have been advanced for targeting different chemokine receptors (56). Despite this, many of them failed to show sufficient clinical responses. In fact, until now, only three chemokine antagonists have been approved for clinical use (57): (i) the CXCR4 antagonist plerixafor, a small molecule that mobilizes hematopoietic stem cells; (ii) the noncompetitive CCR5 antagonist maraviroc, a small molecule that prevents the binding of HIV envelope glycoprotein to CCR5; and (iii) the CCR4 antagonist mogamulizumab, a defucosylated humanized monoclonal antibody approved for the treatment of mycosis fungoides or Sézary syndrome. Some general reasons could explain the treatment failures associated with chemokine receptor antagonists. First, as diseases are associated with many chemokine receptors, blocking only one may not be enough. Second, chemokine receptors take part in many immune and inflammatory activities, so blocking key chemokine receptors may lead to the occurrence of severe adverse events. Third, an effective dosage of non-toxic antagonists, sufficiently metabolically stable in the circulation, is needed to block the chemokine receptor–ligand interactions.

Aggregating the data of 406 patients treated in the various randomized trials performed so far through our meta-analysis, we suggested, for the first time, a significant efficacy of reparixin on a hard endpoint such as the survival. Of note, trials including severely ill patients (lung and kidney transplant recipients, critically ill COVID-19 patients) contributed most to the result. This is not unexpected as interleukin 8 level and neutrophil activation were both previously associated with acute kidney injury and acute respiratory distress syndrome in critically ill patients (37, 58–61).

The meta-analysis results could allow the opportunity not only to identify reparixin as an agent for the treatment of COVID-19-related ARDS (where it is now under evaluation) (62), but also to suggest it as a new therapeutic to treat ARDS of any origin or cause to modulate the inflammatory response and its clinical consequences (63).

Further supporting this is the fact that no safety issues emerged from our meta-analysis, confirming the excellent tolerability profile of reparixin reported in each trial. This was not taken for granted. In fact, it is not possible to exclude *a priori* that an interleukin 8 receptor inhibitor does not increase the risk of infections. Neutrophils play a central role in innate immunity acting as the first line of host defense against infection, and CXCL8 activity is required for neutrophil migration and recruitment to inflamed sites during infection (64, 65). The evidence that the risk of infections is not increased by reparixin treatment supports a complex model of neutrophil recruitment during infection characterized by an early phase, mediated by short-lived signals, and by an amplification phase, which is mediated by signaling cascades through leukotriene-B4 and IL-8/CXCR1-2 pathway (66, 67). Similar to AZD5069 (68), another selective antagonist of CXCR2, the net effect of CXCR2 inhibition by reparixin probably allows neutrophil migration without impacting neutrophil-mediated phagocytic and oxidative burst activities, but preventing the excess neutrophil infiltration and activation (69).

Our study has some limitations that are consistent with meta-analyses of highly heterogeneous studies including differences in target populations, targeted effects, survey recruitment, administration methods, and timing of outcome measurements. Moreover, some of the studies included have a small number of participants and a limited range of age within the studied population.

In conclusion, short-term inhibition of CXCL8 activity with the allosteric inhibitor reparixin improved survival in patients at high risk for in-hospital mortality treated in RCTs. This evidence suggests that the role of IL-8 and its receptors is complex and, overall, clinically relevant. Therapeutic interventions targeting IL-8 receptors in the future should be investigated in critically ill patients with hyperinflammatory complications like ARDS.

## Data availability statement

The raw data supporting the conclusions of this article will be made available by the authors, without undue reservation. Requests to access the data should be directed to landoni.giovanni@hsr.it, piemonti.lorenzo@hsr.it

## Author contributions

All authors participated to ideation, data collection or analysis, and drafting or correcting the manuscript. All authors approved the final version of the manuscript.

## Funding

This manuscript was supported by departmental fund only.

## Acknowledgments

We thank Yuki Kotani (MD), Tóth Krisztina (MD), and Eros Pilia (MD) for carefully revising the manuscript.

## Conflict of interest

The authors declare that the research was conducted in the absence of any commercial or financial relationships that could be construed as a potential conflict of interest.

## Publisher’s note

All claims expressed in this article are solely those of the authors and do not necessarily represent those of their affiliated organizations, or those of the publisher, the editors and the reviewers. Any product that may be evaluated in this article, or claim that may be made by its manufacturer, is not guaranteed or endorsed by the publisher.
