# Supplementary material for: The effect of reparixin on survival in patients at high risk for in-hospital mortality: a meta-analysis of randomized trials
Source: Front Immunol. 2022 Jul 25;13:932251. doi: 10.3389/fimmu.2022.932251 (PMC9358031; doi:10.3389/fimmu.2022.932251)

Supplementary Material

**Index**

Supplementary data P2

Supplementary figures

PRISMA flow diagram P3

Forest plot of the effect of reparixin on mortality removing each study in turn and using the odds ratio with a fixed-effect model P4

Forest plot of the effect of reparixin on mortality in perioperative settings P5

Forest plot of the effect of reparixin on mortality in transplant P6

Forest plot of the effect of reparixin on mortality in studies with the length of treatment ≥ 48 hours P7

Risk of bias analyses P8

Trial sequential analysis (TSA) P9

**Supplementary data**

For the following studies we obtained nonpublished data from the authors (ref A) or from the company (B) that we added to our meta-analysis:

1. Landoni G, Voza A, Puoti M, Coppola N, Akselrod H, Gavioli E, et al. A phase 3 study to evaluate the efficacy and safety of reparixin in severe COVID-19 pneumonia. In: European Respiratory Society (ERS) Congress; 2022 Sep 4-6; Barcellona, Spain.
2. Meyers BF, Keshavjee S, Zamora MR, Davis RD, Smith MA, McFadden PM, et al. 405: A Multicenter Prospective, Randomized, Placebo-Controlled Trial of a CXCL8 Inhibitor (Reparixin) To Prevent Primary Graft Dysfunction after Lung Transplantation. J Heart Lung Transplant. 2008; 27:S206-S207

**Supplementary figures**

**Supplementary Figure 1.** PRISMA flow diagram.

**Supplementary Figure 2.** Forest plot of the effect of reparixin on mortality removing each study in turn and using the odds ratio with a fixed-effect model.

**Supplementary Figure 3.** Forest plot of the effect of reparixin on mortality in perioperative settings.


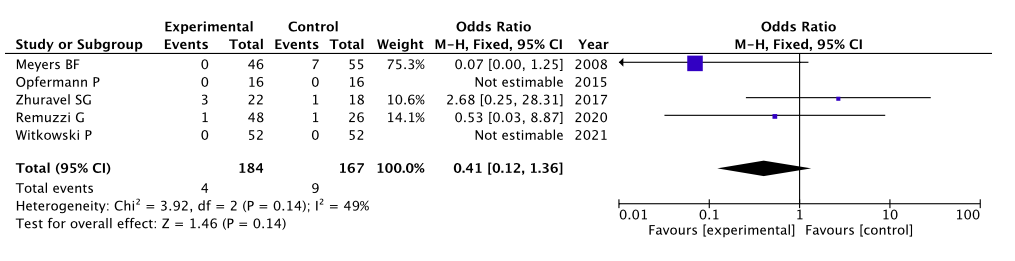


**Supplementary Figure 4.** Forest plot of the effect of reparixin on mortality in transplant.

**Supplementary Figure 5.** Forest plot of the effect of reparixin on mortality in studies with the length of treatment ≥ 48 hours.

**Supplementary Figure 6.** Risk of bias analyses.


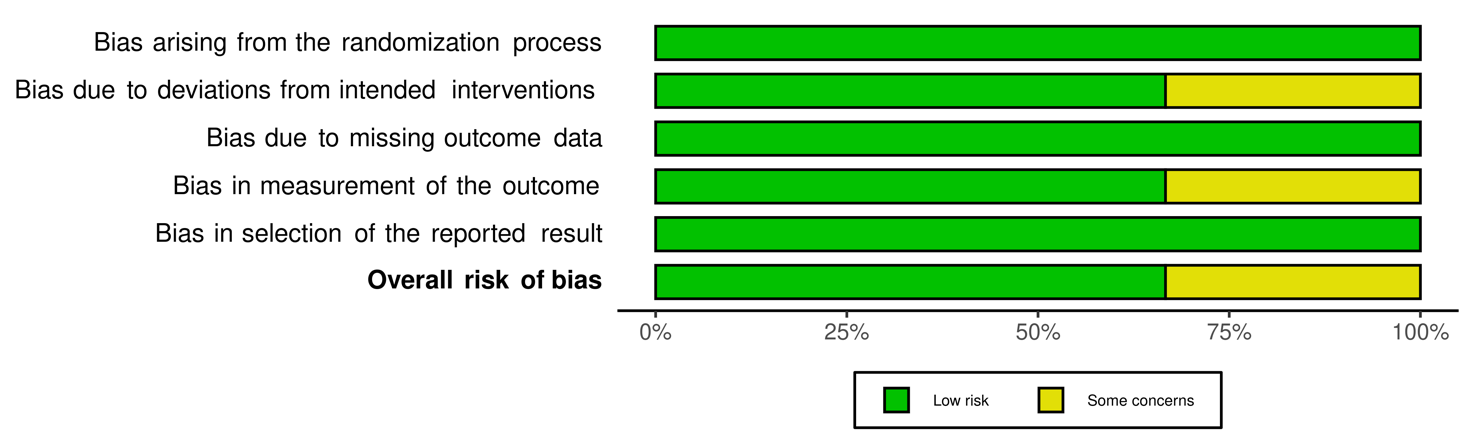


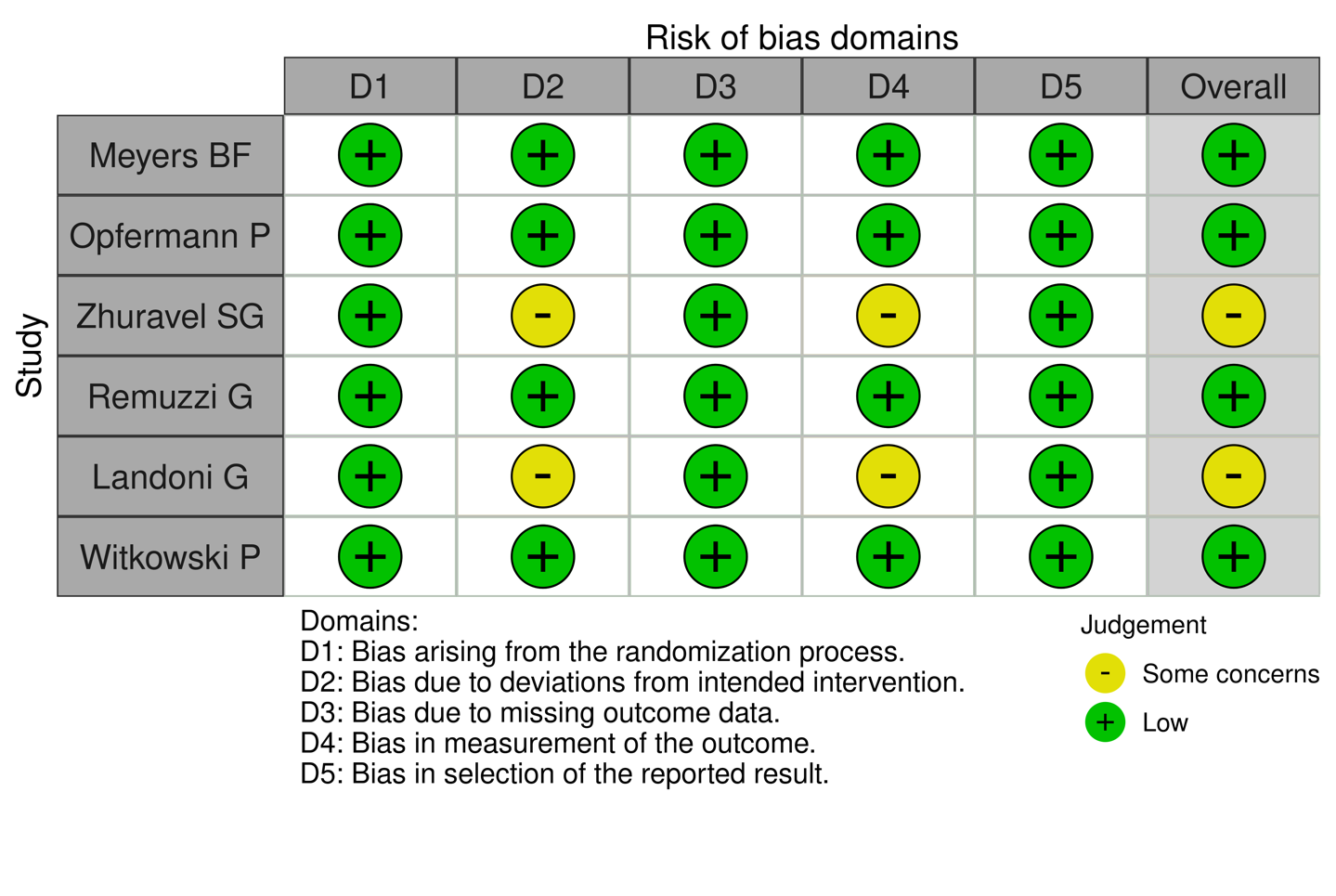


**Supplementary Figure 7.** Trial sequential analysis (TSA).


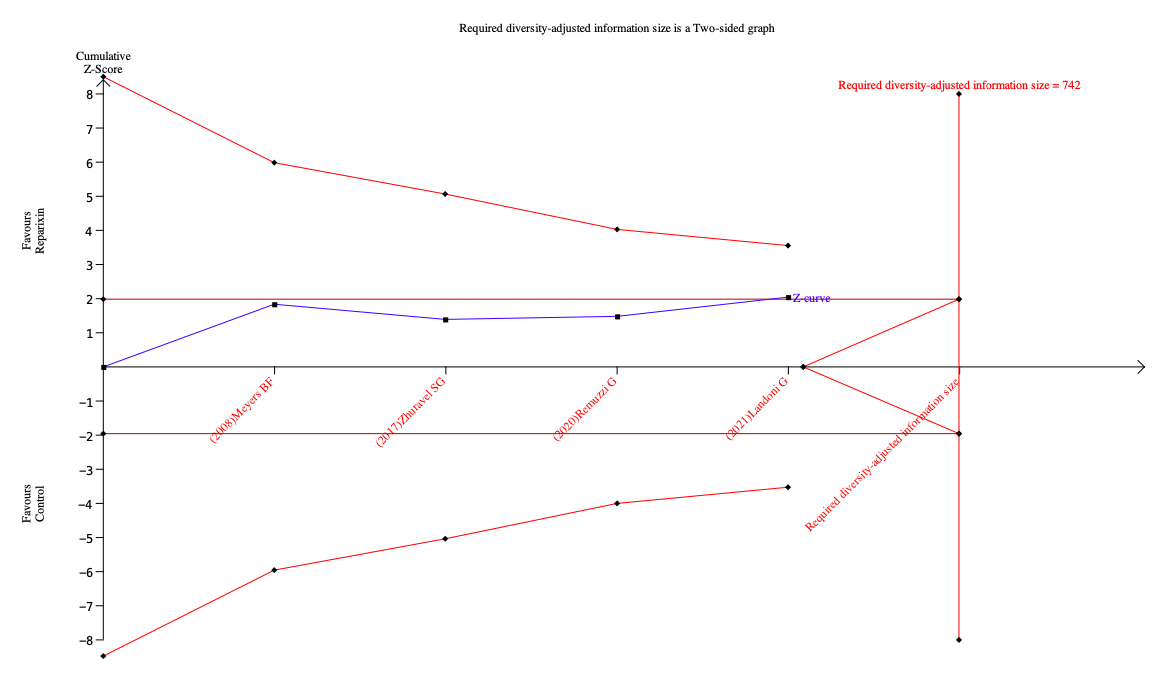

Supplement: Supplementary file 1 [file DataSheet_1.docx]
